# Hypnotherapy, Intermittent Fasting, and Exercise Group Programs in Atopic Dermatitis: A Randomized Controlled Explorative Clinical Trial During the COVID-19 Pandemic

**DOI:** 10.1089/jicm.2022.0699

**Published:** 2023-02-08

**Authors:** Gabriele Rotter, Michael Teut, Romy Schleicher, Melanie Dell'Oro, Miriam Ortiz, Sylvia Binting, Tatjana Tissen-Diabaté, Stephanie Roll, Andreas Michalsen, Doris Staab, Bernd Wolfarth, Benno Brinkhaus

**Affiliations:** ^1^ Department of Epidemiology and Health Economics, Institute of Social Medicine, corporate member of Freie Universität Berlin and Humboldt - Universität zu Berlin, Charité - Universitätsmedizin Berlin, Berlin, Germany.; ^2^ Department of Internal and Integrative Medicine, Immanuel Krankenhaus Berlin, Berlin, Germany.; ^3^ Department of Pediatric Respiratory Medicine, Immunology and Critical Care Medicine, corporate member of Freie Universität Berlin and Humboldt - Universität zu Berlin, Charité - Universitätsmedizin Berlin, Berlin, Germany.; ^4^ Department of Sports Medicine, corporate member of Freie Universität Berlin and Humboldt - Universität zu Berlin, Charité - Universitätsmedizin Berlin, Berlin, Germany.

**Keywords:** atopic dermatitis, hypnosis, fasting, diet, exercise, randomized controlled trial, complementary medicine

## Abstract

**Background:**

Patients with atopic dermatitis (AD) frequently use healthy lifestyle behaviors, although their benefits are unclear. This study's aim was to investigate the effectiveness of hypnotherapy, fasting with diet adjustments, and exercise in AD patients.

**Methods:**

In a four-armed randomized controlled monocenter open explorative clinical trial, adult patients with mild-to-moderate severe AD underwent, over 16 weeks, a five-session hypnotherapy group program (HTP), a five-session intermittent fasting with diet adjustment group program (IFDP), a five-session exercise group program (EP), or no study intervention (control) as add-on to topical corticosteroid use if required. Endpoints included subjectively perceived itching on a visual analogue scale (VAS, 0–100 mm); disease severity by SCORing Atopic Dermatitis (SCORAD); and adverse events (AEs). Endpoints were analyzed descriptively in the Full Analysis Set (FAS). Due to the coronavirus disease 2019 (COVID-19) pandemic, relevant changes to the study protocol included online in addition to “in-presence” group interventions, closing the study arm EP and premature trial termination before randomization of 120 intended patients.

**Results:**

During the COVID-19 pandemic, study recruitment was poor. The FAS included 20 patients (17 female) with 35.0 ± 12.1 (mean ± standard deviation [SD]) years of age. At baseline, mean ± SD for HTP (*n* = 6), IFDP (*n* = 4), EP (*n* = 1), and control (*n* = 9) were VAS itching 63.2 ± 18.0, 65.0 ± 13.9, 43.0 mm, 62.1 ± 17.3; SCORAD 43.0 ± 13.6, 47.0 ± 21.0, 60.3, 39.1 ± 15.6. After 16 weeks, endpoints were VAS itching 26.0 ± 16.4, 31.7 ± 9.9, 23.0 mm, 39.3 ± 27.0; SCORAD 24.1 ± 12.2, 29.1 ± 19.1, 49.1, 25.5 ± 14.4. No serious AEs related to the interventions were observed.

**Conclusion:**

Despite very small groups, study results indicated potential beneficial changes to baseline in perceived itching intensity, disease severity, and disease-specific quality of life for HTP and IFDP. Therefore, further clinical trials should be performed investigating the effectiveness and safety of all interventions.

**Clinical Trial Registration:**

January 31, 2020 German Clinical Trials Register (DRKS): DRKS00020557, Universal Trial Number (UTN): U1111-1247-1512.

## Introduction

Atopic dermatitis (AD) is associated with a high personal, social, and economic burden.^1^ Even first-line symptomatic treatment with topical corticosteroids (TCS) entails a risk of relevant adverse events (AEs).^2,3^ Therefore, patients worry about the treatment and engage in potentially healthy lifestyle behaviors.^4,5^ Relaxation techniques, diet, and exercise can diminish AD symptoms and are recommended.^6–12^ In a Berlin subsample of 58 (50.4% response rate) AD patients, 41.4% engaged in a relaxation technique, 75.9% in nutrition adjustment, and 93.1% in exercise.^13^ Therefore, the authors decided to further investigate related lifestyle behaviors. In animal models, case reports, and in a few mostly nonrandomized clinical trials, they found indications for benefits related to AD for hypnotherapy,^14–18^ intermittent fasting, plant-based or arachidonic acid-restricted food,^19–31^ and exercise.^32–35^

Treatments in a group setting might reduce costs, have beneficial group effects, and may improve quality of life (QoL) and dermatological symptoms.^36,37^ The rationale for this trial was that a hypnotherapy group program (HTP), intermittent fasting with diet adjustment group program (IFDP), and an exercise group program (EP) for outpatients could provide new treatment strategies for AD patients, which could subsequently be implemented in health care practice.

The aim of this study was to exploratively investigate the effectiveness of hypnotherapy, fasting with diet adjustments, and exercise in adult AD patients with a control group, and to gather experience and data for future confirmatory trials.

## Methods

### Study design, setting, and mitigation strategies due to the COVID-19 pandemic

In a four-armed, randomized controlled, single-center, open explorative clinical trial, adult AD patients received HTP, IFDP, EP, or control (nonintervention, waiting list, control). This study followed the standards of the Declaration of Helsinki,^38^ and the ICH-GCP guidelines.^39^ The positive decision of the ethics commission at Charité - Universitätsmedizin Berlin (EA1/315/19) was obtained and the study was registered prospectively (https://www.drks.de/, DRKS00020557). All patients provided oral and written informed consent before their inclusion in the study.

A randomization list was generated by a data manager as a computer-generated block randomization with variable block length. The randomization list was concealed by use of a computer interface, implemented in an MS ACCESS database and managed by the study nurse. The study physician contacted the study nurse by telephone, and after entering participants' inclusion information, participants were assigned to the respective study arm, and randomization results were reported to the study physician. The study interventions HTP and IFDP were performed at the Outpatient Clinic for Integrative Medicine at Campus Mitte Charité - Universitätsmedizin Berlin, EP was performed at the Center for Outpatient Rehabilitation (ZAR), Berlin.

### Changes to the protocol

**FIG. 1.**
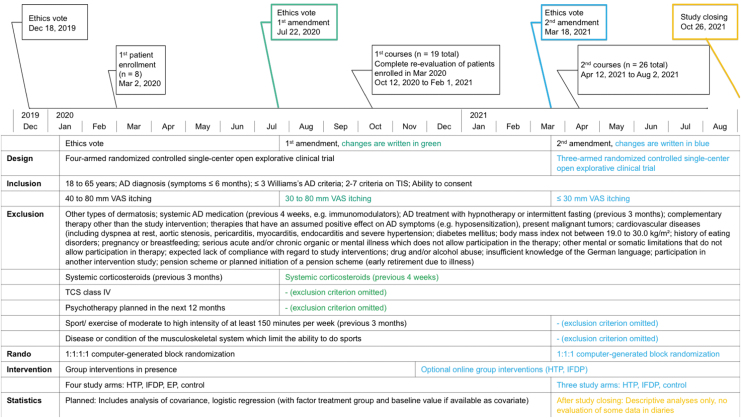
Modifications and Mitigations CAMATOP II. AD, atopic dermatitis; EP, exercise program; HTP, hypnotherapy group program; IFDP, intermittent fasting with diet adjustment group program; TCS, topical corticosteroids class I–III; TIS, three-item severity score; VAS, visual analogue scale.

Since March 2020, the coronavirus disease 2019 (COVID-19) pandemic^40^ seriously impacted clinical research. This study was started at the same time and thus affected by the pandemic-related restrictions. Resulting modifications and mitigations are reported considering the CONSERVE statement (Fig. 1).^41^ *Extenuating circumstances* were changing Berlin's pandemic laws and confinements, with strict in-house regulations prohibiting research “in presence” during many months. *Impacts* as a result included the near-impossible participant recruitment, difficulty in performing physical examination for study inclusion, and “in-presence” endpoint measures or interventions.

*Mitigating strategies* were the constantly updated COVID-19 hygiene concepts, risk–benefit evaluations, and timing adjustments. As a consequence, at the beginning of the study, enrolled patients could not receive their allocated interventions in time. The authors constantly attempted restarts. In October 2020, these patients were re-evaluated by the study physician, and thereafter started together with newly recruited participants, the (now mostly online) group interventions (October 2020 to February 2021). Two ethics commission amendments were obtained for the following: Broadening of inclusion criteria (because of unusually poor recruitment), implementation of online group interventions (HTP and IFDP), recruitment stop, and, by this, closing the study arm EP (because of mandatory “in-presence” exercise monitoring including blood pressure, heart rate).

After passing the foreseen two years for the study project, and with the prospect of renewed stricter research limitations, the study was closed on October 26, 2021. These are important modifications because the protocol changes and the small sample size impaired effectiveness evaluations.

### Patients

Patients were recruited in Berlin by public transport advertisements, posters, and digital media. The inclusion and exclusion criteria with their modifications are provided in Figure 1.

### Study interventions

#### Group intervention programs

In addition to a literature review (trials, therapy books, German Society for Nutrition^42^), stakeholder engagements (six HTP, six IFDP, five EP experts) identified beneficial therapeutic components. Respective experts consented on closed groups with sequential scheduling for group effects. The results were then coordinated for comparability: Each program consisted of five sessions lasting 90 min in groups of 5–10 patients with approximately 2-week intervals. Furthermore, each program (HTP, IFDP, and EP) included supported self-treatments (audio recordings in HTP, a handout with instructions on intermittent fasting with diet adjustments in IFDP, and a handout with exercise instructions in EP). Group interventions were performed by well-educated and clinically experienced hypnotherapists (a physician and a psychologist), one dietician, or two sports scientists, respectively. Each group program was based on intervention-specific health education, training in the intervention, and resource activation. The concepts are displayed in Supplementary Table S1a–c, key characteristics are described below.

#### Hypnotherapy group program

The hypnosis exercises aimed for deep relaxation, increasing well-being and calmness, individual resource activation for skin healing and improving the skin function, strengthening physiological and psychological barrier functions, and to reduce itching.

#### Intermittent fasting with diet adjustment group program

Plant-based food was recommended with at least three daily portions (handful) of vegetables, two portions of fruits, three portions of low-processed cereals. A small amount of animal-derived food was allowed. The time-restricted feeding comprised a 16 (±1)-hour overnight fasting period, with optional modification of 16:8 fasting in one day per week.

#### Exercise group program

Mobility and coordination exercises included games (e.g., with balls, hoops) and walking. A 30-min endurance training complemented each session. The recommended home training combined an intensive-to-moderate endurance training, a moderate strength training (13/20 on a Borg scale),^43^ and stretching exercises of about 150 min per week.

#### Control

The patients could continue their routine care but received no study intervention during the first 16 weeks. Due to COVID-19-derived extenuating circumstances, patients allocated to control could only participate in HTP or IFDP after 26 weeks outside of the study.

Patients in all groups were allowed to use routine care, such as TCS and emollients, but no other AD medication (e.g., immunomodulators, calcineurin inhibitors).

### Endpoints

No primary endpoint was defined, due to the exploratory nature of this trial. The most important endpoint was considered the average subjectively perceived itching intensity within the last seven days measured after 16 weeks on a horizontal visual analogue scale (VAS; 0–100 mm), as it is validated and broadly used in AD trials.^44,45^

The measurements at baseline and after 8, 16, and 26 weeks were as follows: The average subjectively perceived itching intensity within the last seven days on a VAS^44^; the average subjectively perceived total severity of all AD symptoms within the last seven days on a VAS (nonvalidated); number of TCS applications within the last seven days (TCS use, nonvalidated); general QoL using the 12-item Short Form Health Survey (validated)^46–49^; QoL disease-specific by the Dermatology Life Quality Index (DLQI, validated)^50,51^; affects by the Positive and Negative Affect Schedule (validated)^52^; incapacity to work due to AD (hours/during 8 weeks, nonvalidated); the average subjectively perceived sleep disturbance within the last seven days on a VAS (validated)^53^; the average subjectively perceived skin condition within the last seven days on a VAS (nonvalidated).

At baseline and after 8 and 16 weeks, independent raters (physicians and nurses who are neurodermatitis trainers and specialized in SCORing Atopic Dermatitis [SCORAD] rating) performed a group allocation-blinded assessment of the disease severity using the broadly used and validated SCORAD and Eczema Area and Severity Index (EASI).^54,55^

At baseline, patients provided sociodemographic data and rated their expectations regarding study interventions on a numeric rating scale (nonvalidated). After 8, 16, and 26 weeks, patients allocated to HTP, IFDP, or EP reported their perceived efficacy of the respective treatment and all patients reported their self-perceived change of complaints. During the first 16 weeks, all patients recorded their medication and data on compliance to allocated interventions in diaries.

Safety was assessed by AEs recorded in patients' diaries and/or in the study center.

Further details on endpoints (range, minimal clinically important difference) are provided in Table 2.

All data were collected pseudonymously by standardized paper forms and entered in the SoSci Survey.^56^

### Statistical analyses

As this was an explorative clinical trial, no sample size calculation was performed. A total of 120 patients (30 per intervention group) were considered logistically manageable and sufficient to achieve the study's exploratory objectives. Because of insufficient recruitment, the statistical analyses were changed: Instead of analysis of covariance, data were analyzed only descriptively using mean values, standard deviations (SDs), medians, absolute and relative frequencies, and graphically using boxplots. Baseline values were calculated for the whole allocated population and for the Full Analysis Set (FAS) including patients who could be analyzed also at the 8-week follow-up. Missing data were not imputed. Statistical analyses were performed with IBM SPSS for Mac Version 26, IBM SPSS for Windows Version 27.^57^

## Results

**FIG. 2.**
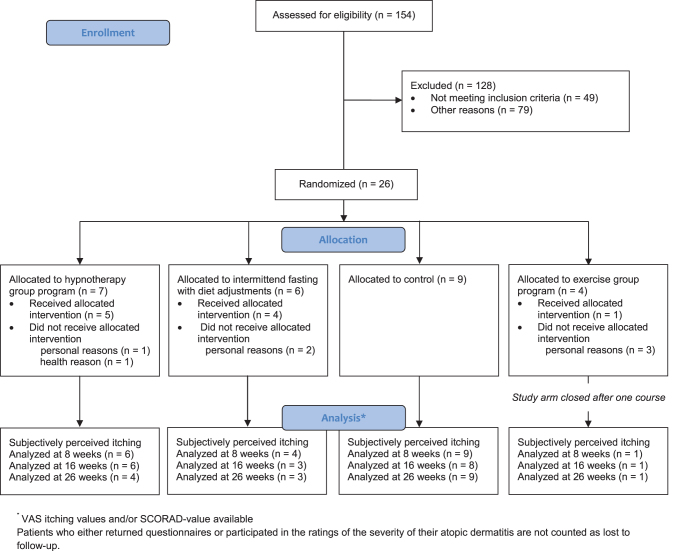
Recruitment, treatment, and follow-up of patients.

Between March 2020 and April 2021, of 154 eligible patients, 26 patients were randomized (HTP *n* = 7, IFDP *n* = 6, EP *n* = 4, control *n* = 9). Because a sample size increase was unlikely due to the COVID-19 pandemic, the study was stopped prematurely. Before the first treatment, five patients dropped out (HTP *n* = 1, IFDP *n* = 1, EP *n* = 3, for further details see Fig. 2). In addition, allocated treatment was not received by one patient randomized to HTP (health reason, AE, data until the follow-up at 16 weeks provided) and another patient randomized to IFDP (personal reason).

The FAS included 20 patients (85.0% female) with 35.0 ± 12.1 (mean ± SD) years of age. The authors found relevant intergroup baseline differences in patients' age, duration of AD disease, number of patients applying TCS during the last four weeks, hours of incapacity to work, and perceived sleep disturbance measured by a VAS (Tables 1 and 2).

**Table 1.** Baseline Characteristics of Participants Who Completed Questionnaires After Week 8 (*n* = 20)

	*N*	HTP (*n* = 6), mean ± SD/*n *(%)	IFDP (*n* = 4), mean ± SD/*n *(%)	Control (*n* = 9), mean ± SD/*n *(%)	EP (*n* = 1), mean ± SD/*n *(%)	Total, mean ± SD/(*n* = 20),* n *(%)
Age (years)	20	29.3 ± 4.0	26.0 ± 12.1	39.6 ± 11.5	56.0	35.0 ± 12.1
Sex (female)	20	6 (100)	3 (75.0)	8 (88.9)	0	17 (85.0)
Sex (male)	20	0	1 (25.0)	1 (11.1)	1 (100)	3 (15.0)
BMI (kg/m^2^)	20	21.4 ± 2.6	21.7 ± 1.4	23.7 ± 3.0	25.2	22.7 ± 2.7
German university entrance qualification	20	6 (100)	3 (75.0)	8 (88.9)	1 (100)	18 (90.0)
Duration of AD (years)	20	22.2 ± 6.4	21.5 ± 15.3	38.1 ± 13.0	54.0	30.8 ± 14.7
Three-item severity score [0–9]^a^	20	4.5 ± 1.0	4.8 ± 2.6	5.9 ± 0.8	5.0	5.2 ± 1.4
Concomitant disease	20	2 (33.3)	2 (50.0)	5 (55.6)	0	9 (45.0)
Allergic asthma	20	2 (33.3)	2 (50.0)	2 (22.2)	0	6 (30.0)
History of allergy	20	6 (100)	2 (50.0)	7 (77.8)	1 (100)	16 (80.0)
Food allergy	20	4 (66.7)	2 (50.0)	3 (33.3)	1 (100)	10 (50.0)
Influence by climate	20	6 (100)	4 (100)	7 (77.8)	1 (100)	18 (90.0)
Previous psychotherapy	20	3 (50.0)	2 (50.0)	8 (88.9)	0	13 (65.0)
Past treatment with hypnotherapy	20	0	0	2 (22.2)	0	2 (10.0)
Due to AD	20	0	0	0	0	0
Due to other diagnosis	20	0	0	2 (22.2)	0	2 (10.0)
Past fasting	20	4 (66.7)	1 (25.0)	5 (55.6)	0	10 (50.0)
Due to AD	20	1 (16.7)	0	2 (22.2)	0	3 (15.0)
Due to other diagnosis	20	3 (50.0)	1 (25.0)	3 (33.3)	0	7 (35.0)
Incapacity to work due to AD (last 8 weeks)	20	3 (50.0)	1 (25.0)	4 (44.4)	1 (100)	9 (45.0)
Sleep disturbances due to AD	20	5 (83.3)	3 (75.0)	6 (66.7)	1 (100)	15 (75.0)
Patient expectations for HTP [0–10]^b^	20	7.2 ± 1.2 Median 7.0	5.8 ± 2.2 Median 6.0	5.1 ± 2.4 Median 5.0	3.0	5.1 ± 2.4 Median 6.0
Patient expectations for IFDP [0–10]^b^	20	5.7 ± 2.3 Median 5.5	5.5 ± 2.4 Median 6.5	6.6 ± 3.2 Median 7.0	4.0	6.0 ± 2.6 Median 7.0
Patient expectations for EP [0–10]^b^	20	3.0 ± 2.5 Median 2.5	5.3 ± 2.2 Median 6.0	5.0 ± 2.5 Median 6.0	2.0	4.3 ± 2.5 Median 4.5

Values are absolute numbers (*N*), column percentages, or mean ± SD.

^a^ Lower values indicate better status.

^b^ 0 = no improvement, 10 = complete recovery.

AD, atopic dermatitis; BMI, body mass index; EP, exercise program; HTP, hypnotherapy group program; IFDP, intermittent fasting with diet adjustment group program; SD, standard deviation.

**FIG. 3.**
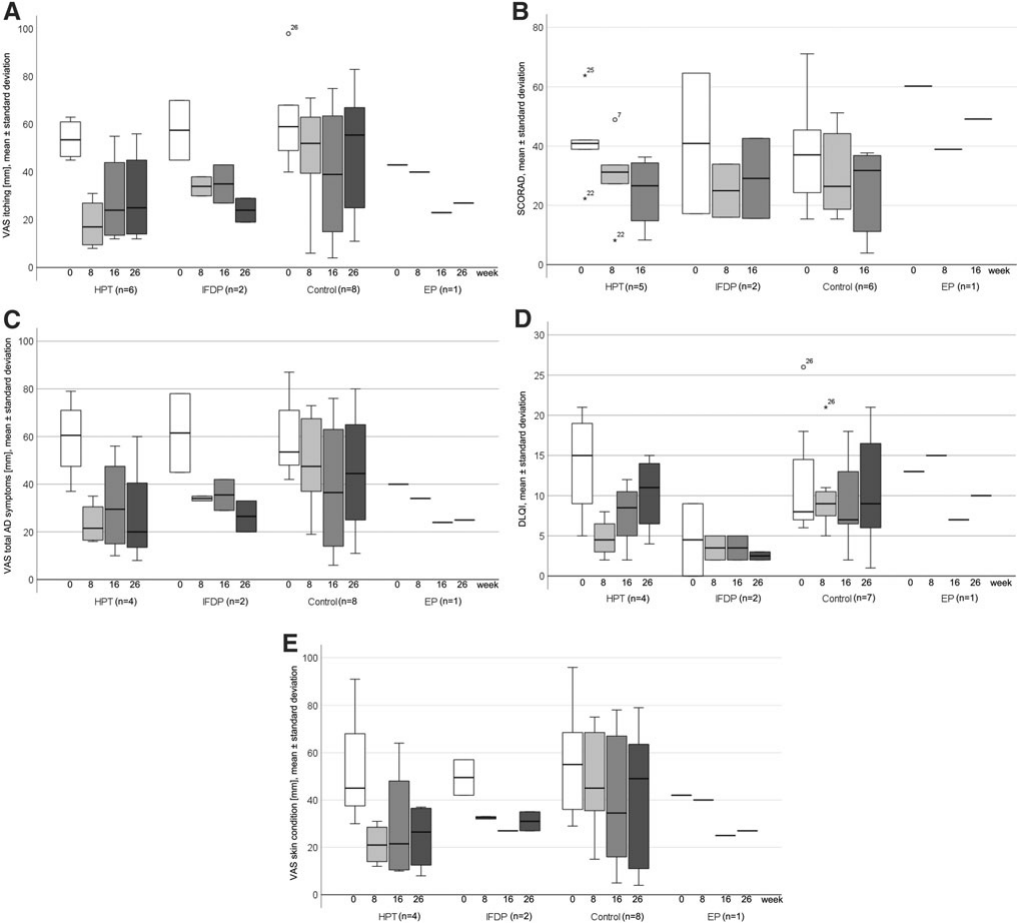
**(A)** Perceived itching intensity on a VAS through 26 weeks in HTP, IFDP, control, and EP. Values are means and standard deviations. **(B)** Disease severity by SCORAD through 26 weeks in HTP, IFDP, control, and EP. Values are means and standard deviations. **(C)** Perceived total severity of all AD symptoms on a VAS through 26 weeks in HTP, IFDP, control, and EP. Values are means and standard deviations. **(D)** Disease-specific quality of life by the DLQI through 26 weeks in HTP, IFDP, control, and EP. Values are means and standard deviations. **(E)** Perceived skin condition on a VAS through 26 weeks in HTP, IFDP, control, and EP. Values are means and standard deviations. AD, atopic dermatitis; DLQI, Dermatology Life Quality Index; EP, exercise group program; HTP, hypnotherapy group program; IFDP, intermittent fasting with diet adjustment group program; SCORAD, SCORing Atopic Dermatitis; VAS, visual analogue scale.

In the comparison of baseline with 8 or 16 weeks, the authors observed indications for beneficial effects in perceived itching intensity on VAS, disease severity by SCORAD, perceived total severity of all AD symptoms on VAS, disease-specific QoL by DLQI, and perceived skin condition on VAS for HTP, IFDP, and smaller for control (Table 2; Fig. 3A–E). The medication regimens were comparable between groups over time.

**Table 2.** Baseline Values and Endpoints After 8, 16, and 26 Weeks (Patients Who Completed Questionnaires After Week 8), *n* = 20

Endpoint		HTP (*n* = 6), mean ± SD/*n *(%)		IFDP (*n* = 4), mean ± SD/*n *(%)		Control (*n* = 9), mean ± SD/*n *(%)		EP (*n* = 1), mean ± SD/*n *(%)
VAS itching [0–100 mm],^a^ MCID 13.4								
Baseline	6	63.2 ± 18.0	4	65.0 ± 13.9	9	62.1 ± 17.3	1	43.0
8 Weeks	6	25.0 ± 15.2	4	30.3 ± 19.2	9	49.0 ± 19.7	1	40.0
16 Weeks	6	26.0 ± 16.4	3	31.7 ± 9.9	8	39.3 ± 27.0	1	23.0
26 Weeks	4	29.5 ± 20.1	3	40.0 ± 28.2	9	48.1 ± 24.5	1	27.0
SCORAD [0–103],^b^ MCID 8.7								
Baseline	6	43.0 ± 13.6	4	47.0 ± 21.0	9	39.1 ± 15.6	1	60.3
8 Weeks	6	32.9 ± 15.0	4	22.7 ± 7.8	9	32.2 ± 15.5	1	38.9
16 Weeks	5	24.1 ± 12.2	2	29.1 ± 19.1	6	25.5 ± 14.4	1	49.1
EASI [0–72],^b^ MCID 6.6								
Baseline	6	12.4 ± 11.0	4	12.4 ± 7.6	9	10.6 ± 8.0	1	28.5
8 Weeks	6	7.4 ± 5.8	4	4.6 ± 4.9	9	9.1 ± 8.6	1	18.9
16 Weeks	5	4.7 ± 3.9	2	5.4 ± 6.5	6	7.6 ± 6.8	1	35.7
VAS total AD symptoms [0–100 mm],^c^ no MCID								
Baseline	6	65.7 ± 18.8	4	72.5 ± 19.5	9	59.6 ± 14.7	1	40.0
8 Weeks	6	28.0 ± 9.9	4	31.8 ± 17.8	9	48.4 ± 19.9	1	34.0
16 Weeks	6	27.8 ± 17.0	3	32.7 ± 8.1	8	38.6 ± 27.2	1	24.0
26 Weeks	4	27.0 ± 22.7	3	40.7 ± 25.4	9	45.1 ± 22.9	1	25.0
Patients using AD medication (systemic or topic)								
Baseline (last 3 months)	6	5 (83.3)	4	4 (100)	9	9 (100)	1	1 (100)
8 Weeks (last 8 weeks)	6	4 (66.7)	4	4 (100)	9	9 (100)	1	1 (100)
16 Weeks (last 8 weeks)	6	4 (66.7)	3	3 (100)	8	6 (75.0)	1	1 (100)
26 Weeks (last 8 weeks)	4	2 (50.0)	3	3 (100)	9	9 (100)	1	1 (100)
Patients using TCS								
Baseline (last 4 weeks)	5	2 (40.0)	4	2 (50.0)	9	8 (88.9)	1	1 (100)
8 Weeks (last 8 weeks)	4	1 (0.25)	3	2 (66.7)	8	7 (87.5)	1	1 (100)
16 Weeks (last 8 weeks)	4	0	3	0	6	5 (83.3)	1	1 (100)
26 Weeks (last 8 weeks)	2	0	3	2 (66.7)	9	7 (77.8)	1	1 (100)
SF-12 PCS,^d^ MCID 5								
Baseline	6	49.8 ± 3.7	4	52.1 ± 8.5	8	50.6 ± 7.8	1	57.9
8 Weeks	6	53.1 ± 7.8	4	52.5 ± 5.4	9	52.0 ± 7.5	1	41.7
16 Weeks	6	55.0 ± 6.5	3	53.3 ± 3.2	8	50.2 ± 6.5	1	46.8
26 Weeks	4	56.8 ± 6.6	2	49.3 ± 9.4	9	52.3 ± 7.6	1	58.3
SF-12 MCS,^d^ MCID 5								
Baseline	6	42.7 ± 4.3	4	43.7 ± 9.8	8	40.9 ± 9.1	1	39.3
8 Weeks	6	36.3 ± 7.5	4	51.1 ± 4.0	9	43.0 ± 6.4	1	29.6
16 Weeks	6	40.9 ± 7.8	3	50.9 ± 4.4	8	40.1 ± 7.3	1	50.0
26 Weeks	4	35.1 ± 2.5	2	53.4 ± 9.3	9	33.3 ± 8.5	1	33.7
DLQI [0–30],^b^ MCID 3.3								
Baseline	6	12.8 ± 6.3	4	11.8 ± 11.7	9	11.3 ± 6.8	1	13.0
8 Weeks	6	6.0 ± 2.8	4	5.3 ± 4.7	8	9.9 ± 4.9	1	15.0
16 Weeks	6	7.0 ± 4.1	3	5.7 ± 4.0	7	9.4 ± 5.6	1	7.0
26 Weeks	4	10.3 ± 4.9	3	9.0 ± 11.3	9	9.6 ± 7.1	1	10.0
PANAS, positive affect dimension [1–5]^b^								
Baseline	6	2.9 ± 0.2	4	3.2 ± 0.4	9	2.7 ± 0.9	1	3.1
8 Weeks	6	2.9 ± 0.6	4	3.8 ± 0.2	9	2.7 ± 0.7	1	2.5
16 Weeks	6	3.1 ± 0.6	3	3.6 ± 0.5	8	2.7 ± 0.7	1	2.6
26 Weeks	4	3.0 ± 0.7	3	3.5 ± 0.7	9	2.4 ± 0.4	1	2.9
PANAS, negative affect dimension [1–5]^d^								
Baseline (8 weeks)	6	2.3 ± 0.8	4	1.9 ± 0.2	9	2.0 ± 0.5	1	1.6
8 Weeks	6	2.0 ± 0.6	4	1.9 ± 0.5	9	1.8 ± 0.4	1	1.7
16 Weeks	6	2.0 ± 0.3	3	1.6 ± 0.3	8	2.0 ± 0.4	1	2.0
26 Weeks	4	2.2 ± 0.3	2	1.5 ± 0.3	9	2.0 ± 0.4	1	1.5
Incapacity to work due to AD (hours/during 8 weeks)								
Baseline (yes *n* = 9)	6	41.7 ± 67.8	1	96	9	55.5 ± 61.5	1	8
8 Weeks (yes *n* = 9)	3	114.0 ± 192.3	1	120.0	3	129.3 ± 220.6	1	34.0
16 Weeks (yes *n* = 9)	3	18.3 ± 25.7	1	26.0	4	97.3 ± 101.0	1	8.0
26 Weeks (yes *n* = 8)	1	4.0	1	120.0	5	136.2 ± 121.8	1	10.0
VAS sleep disturbances [0–100 mm],^e^ MCID 10								
Baseline	5	34.8 ± 19.9	3	57.7 ± 21.0	6	46.0 ± 20.9	1	19.0
8 Weeks (yes *n* = 11)	3	16.0 ± 12.1	2	32.5 ± 6.4	5	35.2 ± 27.1	1	19.0
16 Weeks (yes *n* = 8)	1	31.0	3	17.0 ± 12.3	4	39.5 ± 39.2		
26 Weeks (yes *n* = 14)	4	14.8 ± 11.7	3	28.7 ± 38.6	6	29.0 ± 29.4	1	18.0
VAS skin condition [0–100 mm],^f^ no MCID								
Baseline	6	58.7 ± 27.3	4	63.3 ± 19.6	9	55.9 ± 21.4	1	42.0
8 Weeks	6	27.2 ± 11.8	4	29.0 ± 18.0	9	49.0 ± 19.5	1	40.0
16 Weeks	6	28.7 ± 19.6	3	23.7 ± 5.8	8	39.8 ± 28.3	1	25.0
26 Weeks	4	24.5 ± 14.3	3	45.0 ± 24.6	9	40.3 ± 27.4	1	27.0
Patient efficacy (HTP, IFDP, EP)								
8 Weeks	6		4				1	
Very effective		0		1 (25.0)				0
Effective		4 (66.7)		2 (50.0)				0
Less effective		2 (33.3)		1 (25.0)				1 (100)
Ineffective		0		0				0
16 Weeks	6		3				1	
Very effective		0		1 (33.3)				0
Effective		2 (33.3)		1 (33.3)				0
Less effective		3 (50.0)		1 (33.3)				1 (100)
Ineffective		1 (16.7)		0				0
26 Weeks	4		3				1	
Very effective		0		1 (33.3)				0
Effective		1 (25.0)		0				0
Less effective		3 (75.0)		0				1 (100)
Ineffective		0		2 (66.7)				0
Change of complaints								
8 Weeks	6		4		9		1	
Very much improved		0		1 (25.0)		0		0
Greatly improved		4 (66.7)		2 (50.0)		1 (11.1)		0
Minimally improved		1 (16.7)		0		1 (11.1)		0
Not changed		0		1 (25.0)		4 (44.4)		1 (100)
Minimally worsened		0		0		3 (33.3)		0
Very much worsened		1 (16.7)		0		0		0
16 Weeks	6		3		8		1	
Very much improved		0		0		0		0
Greatly improved		3 (50.0)		1 (33.3)		1 (12.5)		0
Minimally improved		2 (33.3)		2 (67.7)		1 (12.5)		1 (100)
Not changed		1 (16.7)		0		4 (50.0)		0
Minimally worsened		0		0		2 (25.0)		0
Very much worsened		0		0		0		0
26 Weeks	4		3		9		1	
Very much improved		0		0		0		0
Greatly improved		1 (25.0)		0		0		0
Minimally improved		0		1 (33.3)		1 (11.1)		0
Not changed		2 (50.0)		2 (67.7)		7 (77.8)		1 (100)
Minimally worsened		1 (25.0)		0		1 (11.1)		0
Very much worsened		0		0		0		0

Values are absolute numbers (*N*), column percentages or mean ± SD.

^a^ 0 = no itching, 100 = extreme itching.

^b^ Lower values indicate better status.

^c^ 0 = no symptoms, 100 = extreme symptoms.

^d^ Higher values indicate better status.

^e^ 0 = no sleep disturbance, 100 = extreme sleep disturbance.

^f^ 0 = undisturbed skin, 100 = extremely disturbed skin.

AD, atopic dermatitis; DLQI, Dermatology Life Quality Index; EASI, Eczema Area and Severity Index; EP, exercise program; HTP, hypnotherapy group program; IFDP, intermittent fasting with diet adjustment group program; MCID, minimal clinically important difference; MCS, mental component scale; PANAS, Positive and Negative Affect Schedule; PCS, physical component scale; SCORAD, SCORing Atopic Dermatitis; SD, standard deviation; SF-12, 12-item Short Form Health Survey; TCS, topical corticosteroids class I–III; VAS, visual analogue scale.

Regarding safety, one serious AE (SAE) was reported by one patient allocated to IFDP (AD symptom worsening, hospitalization before study intervention start) and one patient allocated to control (psychological symptoms, hospitalization). One patient allocated to HTP reported AD symptom worsening (AE) after the first and second session, with unsure relation to HTP. The patient used a topical immunomodulator (pimecrolimus) during weeks 1–3, which managed most symptoms. The patient provided data until the 16-week follow-up, and in additional interviews she reported persisted worsening of symptoms.

The authors found no anamnestic red flag or indication as to why this patient reacted the way she did. Another patient reported short duration of increased itching and psychological symptoms directly after HTP (by patient-related AE) in diary entries at weeks 1 and 3. One patient allocated to IFDP reported skin impurities and digestive problems (by patient-related AE) at week 2. The patient allocated to EP reported no AE.

Regarding feasibility, one HTP and one IFDP “in-presence” session (October 2020) and the subsequent online group meetings (HTP, IFDP) were feasible, but therapists reported to the study coordinator impaired perceived group feelings. At the beginning of the respective fifth sessions, the therapists got positive feedback regarding performance, comfort of online meetings for patients not living in Berlin, and perceived overall study effects. Patients allocated to HTP mostly applied self-hypnotherapy and used other relaxation methods. Patients allocated to IFDP reported mostly good adherence to the 16:8 fasting protocol. The EP was reduced to a single setting program joining in-house rehabilitation patients with five sessions. Therapists reported good exercise and home exercise feasibility, and patients' symptom reduction. Data of these patients are provided in tables and figures without further comments. Baseline data for the whole included sample are provided in Supplementary Table S2.

## Discussion

The CAMATOP II trial was strongly impacted by confinements and research restrictions due to the COVID-19 pandemic. However, in the 20 included AD patients, the authors observed partly relevant beneficial changes over time in perceived itching intensity, disease severity, and disease-specific QoL for HTP, IFDP, and partly also for control. One AE with aggravation of itching and eczema with the use of a topical immunomodulator was unsurely related to HTP; other AEs by patients related to HTP or IFDP were minor and short-lived, probably by focusing the patients' attention on the skin. Reported SAEs were not related to the interventions. The HTP and IFDP “in-presence” and online meetings were feasible and can be utilized for further research. The “in-presence” EP was only applied to a single AD patient, but a group setting seemed feasible. In online meetings, special group feelings were not perceived by therapists, but patients gave positive feedback.

The COVID-19 pandemic meant that substantial modifications of the study methodology were required. Participant recruitment was insufficient despite multiple advertisements. In a former trial including adult AD patients,^58^ Berlin public transport advertisements were the most effective recruitment strategy. During the COVID-19 confinements, this advertisement strategy was ineffective, as people avoided public transport. In addition, following the experience in study recruitment, the months October to April were expected to be the best time for participant inclusion, but during these months in 2019/2020 and 2020/2021, the COVID-19 pandemic waves in Germany and the ensuing research restrictions were at their height. Mitigating strategies (protocol changes) were applied to enable recruitment and study performance. The closed-group design could have been an obstacle if more AD patients would have been interested in the study.

For larger studies, the authors advise different parallel sessions for each intervention. Finally, the study project could not be performed within the foreseen two-year period and was closed facing renewed research restrictions. From an ethical point of view, results of even few participants should be published to contribute to the scientific evidence. This applies especially for this study, as patients and the study team invested multiple extra efforts.

To our knowledge, this was the first study investigating group programs in hypnotherapy, intermittent fasting with diet adjustments, and exercise in AD patients compared with a nonintervention control, gathering experience and first data. Intergroup comparisons could not be performed. However, the use of hypnotherapy in atopic diseases has only been tested in few exploratory studies, and very rarely within the past 20 years.^14–18^ Senser et al (2004) randomized 33 adult AD patients to 12 single hypnotherapy sessions á 1 h, or to the nonintervention control.^14^ Patients allocated to hypnotherapy were reported to show improvements pre–post and in comparison with control patients regarding the severity of AD by SCORAD, subjectively perceived itching intensity (VAS), subjectively perceived skin condition (VAS), and QoL disease-specifically by the DLQI.^14^ The authors interpreted that 12 sessions of single hypnotherapy is an effective treatment. This study reported no AE. Earlier trials did not report AEs.^59^

In contrast, and in addition to minor AEs, in this study there was one AE unsurely related to HTP with worsening of symptoms (using a topical immunomodulator). The authors found no other explanation for the individual worsening. However, the possibility of symptom worsening is important for patient information and shared decision-making in hypnotherapy. Delaitre et al performed a retrospective data analysis in 27 AD outpatients (mean age 34.5 years) with symptom persistence despite local AD therapy treated with 2–16 individual hypnotherapy sessions á 20 min. The authors found a reduction in disease severity by EASI (measured by the treating physician) and patients' self-assessments, but reported no AE assessment. They concluded that hypnotherapy might be useful in AD patients.^60^ Regarding nutrition, single-armed trials reported AD symptom improvements after time-restricted feeding with a low-energy diet,^21^ and after a calorie-reduced vegan diet.^22^

The authors found no further clinical trials in adult AD patients. Although they found the medication regimens comparable between groups, they could not correlate endpoints to medication use, and therefore, they cannot exclude that medication regimens could have influenced the clinical endpoints. The authors can draw no conclusions from the one patient allocated to EP, as outcome changes might be by chance as in prospective case reports.

For the first time, group interventions such as HTP, IFDP, and EP were developed for adult AD patients in a university outpatient setting “in-presence” and as online group interventions. The strengths of this study include the implementation of blinded-rated and patient-reported endpoints, and the reporting of mitigations due to the COVID-19 pandemic based on the CONSERVE statement. The main limitations were caused by the COVID-19 pandemic. The change in the trial design (closing one study arm) and the small sample size substantially impair the effectiveness evaluations. However, as this is an exploratory trial, the feasibility of the study interventions could be investigated to a certain extent and the interventions proved practicable, as “in-presence” and as online meetings.

Further study limitations include the monocenter setting and the female predominance, which further limits the generalizability of the results. The study design had potential sources of bias: Patients performing HTP, IFDP, or EP received more therapist and/or peer group time and attention than control patients, and blinding of patients and therapists was impossible. Consequently, the observed indications for improvements may have been overestimated. Emerging trends should be verified by confirmatory studies. Further studies should consider to use the subjectively perceived itching intensity (VAS) as a primary endpoint, as this is to patients a meaningful and validated measurement.

Even with a modified study design, the study results are meaningful as they provide data and methodology for the group interventions, in presence and online. As patients participated in studies during the COVID-19 pandemic, they think it is ethical to publish the results. They provided an example, how to report a changed study design and results according to the CONSERVE statement.

The study results suggest that study interventions are feasible, acceptable, and might be effective, providing justification for the progression to confirmatory clinical studies on the interventions. For larger clinical trials, the authors advise different parallel sessions for each intervention, so that patients can choose convenient timeslots.

## Conclusion

The study interventions were feasible and acceptable for patients with mild-to-moderate severe AD, however, the recruitment was difficult due to the COVID-19 pandemic. Despite very small groups, study results indicated potential beneficial changes to baseline in perceived itching intensity, disease severity, and disease-specific QoL for HTP and IFDP. Therefore, further high-quality clinical trials should be performed investigating the effectiveness and safety of hypnotherapy, fasting with diet adjustments, as well as exercise.

## Supplementary Material

Supplemental data

Supplemental data

Supplemental data

## Data Availability

The data sets used and/or analyzed during the current study are not publicly available due to concerns that the individual privacy of the participants could be compromised.
