# Supplementary material for: Hypnotherapy, Intermittent Fasting, and Exercise Group Programs in Atopic Dermatitis: A Randomized Controlled Explorative Clinical Trial During the COVID-19 Pandemic
Source: J Integr Complement Med. 2023 Feb 8;29(2):99–110. doi: 10.1089/jicm.2022.0699 (PMC9942184; doi:10.1089/jicm.2022.0699)
Supplement: Supplemental data [file Suppl_TableS1.docx]

**Supplement Table 1a.** Concept hypnotherapy group program in the CAMATOP II study.

| **Sessions** | Procedure | Duration  (minutes) |
| --- | --- | --- |
| **1. Session** | Getting acquainted, promotion of group cohesion and rapport, naming/identifying one’s own strength | 10 |
|  | **Health Education on atopic dermatitis and management with hypnotherapy, "seeding"**: psychological possibilities of influence, mind-body connection, stress reduction, skin healing, and interrupting itching and scratching with hypnosis, enhancement of well-being overall and on the skin, need for continuous practice through self-hypnosis with the provided audio recordings | 20 |
|  | **Hypnosis: Place of health**  - Induction: eye fixation  - Breath observation  - 0-10 counting  - Perceive and enjoy place of health with all 5 senses  - Suggestions for well-being, refueling, self-healing, skin healing  - Posthypnotic suggestion  - Walking back stairs  - Reorientation | 30 |
|  | **Feedback** (experiences and discussion, utilization and motivation of positive experiences) | 20 |
|  | **Instruction for self-hypnosis practice of session 1 and farewell** (internet link, best to practice once per day and as needed and desired) | 10 |
| **2. Session** | **Exploration of the experiences with practicing independently at home** | 10 |
|  | **Health education:** resources for atopic dermatitis, internal and external resources | 20 |
|  | **Hypnosis: Resource activation**  **-** Induction: eye fixation  - Breath observation  - Stair steps  - Visualize resources  - Resource symbol  - Adjust resource experience  - Mirror exercise (self-image)  - Resource transfer  - Suggestions for well-being, refueling, self-healing, skin healing  - Posthypnotic suggestion  - Walking back stairs  - Reorientation | 30 |
|  | **Feedback** (experiences and discussion, utilization and motivation of positive experiences) | 20 |
|  | **Instruction for self-hypnosis practice of session 2 and farewell** | 10 |
| **3. Session** | **Exploration of the experiences with practicing independently at home** | 10 |
|  | **Health education:** Rapid activation of resources in three short self-hypnosis steps | 20 |
|  | **1. Resource activation and resource transfer**  Hypnosis 1:  - 10-0 counting  - Resource activation through symbol  - Flowing into bowls into hands  - Resource transfer to body  - Posthypnotic suggestion  - 0-10 counting reorientation  **2. Short form of Hypnosis 1**  Hypnosis 2: Hypnosis 1 quick run through  **3. Interrupt itching with coolness**  Hypnosis 3:  - 10-0 counting  - Imagine coolness  - Resource transfer  - Posthypnotic suggestion  - 0-10 counting reorientation | 45 |
|  | **Feedback after each hypnosis** (experiences and discussion, utilization and motivation of positive experiences) | 10 |
|  | **Instruction for self-hypnosis practice of session 3 and farewell** | 10 |
| **4. Session** | **Exploration of the experiences with practicing independently at home** | 10 |
|  | **Health education:** Boundaries, function of the skin as a boundary surface | 20 |
|  | **Hypnosis: Boundaries**  - 10-0 counting  - Text on borders and boundaries  - 0-10 counting reorientation | 30 |
|  | **Feedback after each hypnosis** (experiences and discussion, utilization and motivation of positive experiences) | 10 |
|  | **Instruction for self-hypnosis practice of session 4 and farewell** | 10 |
| **5. Session** | **Exploration of the experiences with practicing independently at home** | 10 |
|  | **Health education:** Integration of the experiences of the last weeks in the unconscious mind | 20 |
|  | **Hypnosis: Healing bath**  - Induction: eye fixation  - Breath observation  - 0-10 counting  - Taking a healing bath in water (open suggestion)  - Enjoying with all senses, especially feeling the sensations on skin  - Plant metaphor  - Suggestions for strengthening the well-being, refueling, self-healing, skin healing, strengthening the skin barrier, nourishment, immunological balance and balance, against itching  - Future progression  - Posthypnotic suggestion  - Walking back stairs  - Reorientation | 30 |
|  | **Feedback after each hypnosis** (experiences and discussion, utilization and motivation of positive experiences) | 10 |
|  | **Instruction for self-hypnosis practice of session 5 and farewell** | 10 |

**Supplement Table 1b.** Concept intermittent fasting and diet modification group program in the CAMATOP II study.

| **Sessions** | **Procedure** | **Duration**  **(minutes)** |
| --- | --- | --- |
| **1. Session** | Getting acquainted, promotion of group cohesion and rapport | 15 |
|  | Health Education on atopic dermatitis and management (Basic information on nutrition and immune system/skin, interval fasting, anti-inflammatory diet.) | 45 |
|  | Questions and answers | 15 |
|  | Instruction for intermittent fasting and diet modification in everyday life (Reflection on nutritional behavior with the help of diaries, handing out diaries.) | 10 |
|  | Farewell | 5 |
| **2. Session** | Exploration of the experiences (Reinforcement, positive experiences and motivation) | 30 |
|  | Health Education (Information on fats and proteins, recipes for food preparation.) | 45 |
|  | Questions and answers | 15 |
|  | Instruction for intermittent fasting and diet modification in everyday life (Deepening of the topic, handouts on the topic) | 10 |
|  | Farewell | 5 |
| **3. Session** | Exploration of the experiences (Reinforcement, positive experiences and motivation) | 30 |
|  | Health Education (Information about carbohydrates, drinks, recipes for food preparation.) | 45 |
|  | Questions and answers | 15 |
|  | Instruction for intermittent fasting and diet modification in everyday life (Deepening of the topic, handouts on the topic.) | 10 |
|  | Farewell | 5 |
| **4. Session** | Exploration of the experiences (Reinforcement, positive experiences and motivation) | 30 |
|  | Health Education (Information on the subject of secondary plant substances, vital substances, recipes for food preparation.) | 45 |
|  | Questions and answers | 15 |
|  | Instruction for intermittent fasting and diet modification in everyday life (Deepening of the topic, handouts on the topic) | 10 |
|  | Farewell | 5 |
| **5. Session** | Exploration of the experiences (Reinforcement, positive experiences and motivation) | 30 |
|  | Health Education (Information on the topic of nutritional psychology (influence of enjoyment, mindfulness and stress on eating behavior and metabolism), practice exercises) | 45 |
|  | Questions and answers | 15 |
|  | Instruction for intermittent fasting and diet modification in everyday life (Deepening of the topic, handouts on the topic.) | 10 |
|  | Farewell | 5 |

**Supplement Table 1c.** Concept exercise group program in the CAMATOP II study.

| **Sessions** | **Procedure** | **Duration**  **(minutes)** |
| --- | --- | --- |
| **1. Session** | **Balls in different sizes and clear the clubs**  *Location: Rehabilitation center, gymnastics room, size of the playing field approximately 4 x 4 m* |  |
|  | Getting acquainted, promotion of group cohesion and rapport | 5 |
|  | Health Education on atopic dermatitis and exercise | 10 |
|  | Ball swap exercises in the group, *Equipment: Gymnastic balls of different making, softballs (ø 10 cm), pezzi ball (ø 55 cm)*  Game: Clear the clubs, in two teams, *Equipment: Bench, clubs, gymnastic balls (ø 15 cm)* | 30 |
|  | Stretching exercise, instruction for self-exercise | 10 |
|  | Farewell (handing out instructions for self-exercise) | 5 |
|  | **Endurance training** (bicycle ergometer, treadmill, walker, etc.), before or after group exercise  - Initial load for women: 1.5 watts/kg body weight, initial load for men: 1.8 watts/kg body weight  - Borg scale: approximately 13/20, controlled by heart rate, blood pressure | 30 |
| **2. Session** | **Nordic walking**  *Meeting point: Rehabilitation center, exercise location: Park at the Nordbahnhof*  *Equipment: walking sticks* |  |
|  | Exploration of the experiences | 5 |
|  | Going to the park at the Nordbahnhof together | 8 |
|  | Warm-up gymnastics with walking sticks | 5 |
|  | Technique training including adjustment of stick lengths | 9 |
|  | Nordic Walking in practice under supervision | 15 |
|  | Stretching exercise, instruction for self-exercise | 5 |
|  | Going back to the rehabilitation center together | 8 |
|  | Farewell | 5 |
|  | **Endurance training** (bicycle ergometer, treadmill, walker, etc.), before or after group exercise  Load build-up individually, monitoring via heart rate, blood pressure and Borg scale | 30 |
| **3. Session** | **Hoops**  *Location: Rehabilitation center, gymnastics room, size of the playing field approximately 4 x 4 m* |  |
|  | Exploration of the experiences | 10 |
|  | Exercises and games with hoops  *Equipment: hoops, bean bags a 200g*  - Hoop gymnastics in place (whole body workout, mobilization and coordination exercises).  - Movement game: "Came alone"  - Rolling hoops to a partner, target throwing at tyre pyramid lying on the ground with bean bags | 30 |
|  | Stretching exercise, instruction for self-exercise | 15 |
|  | Farewell | 5 |
|  | **Endurance training** (bicycle ergometer, treadmill, walker, etc.), before or after group exercise  Load build-up individually, monitoring via heart rate, blood pressure and Borg scale | 30 |
| **4. Session** | **Parachutes**  *Location: Rehabilitation center, gymnastics room, size of the playing field approximately 4 x 4 m* |  |
|  | Exploration of the experiences | 10 |
|  | Exercises and games with a parachute  *Equipment: Circular parachute made of light plastic (ø3m) with approx. 8 cm center hole, water polo ball (ø15 cm), small softball.*  - group in circle around the parachute: parachute shakings in group; parachute slow up/down (synchronic to breathing); changing places by walking fast under the parachute when it is swinging straight up; "laola wave"; rotating a water polo ball on the parachute  - Game: "cat and mouse" (2 teams) cat: water ball, mouse: small softball  - Game: "Ball chasing" in 2 teams, *Equipment: Gymnastic balls, one small exercise ball (synonyms medicine ball, fitness ball)* | 30 |
|  | Stretching exercise, instruction for self-exercise | 15 |
|  | Farewell | 5 |
|  | **Endurance training** (bicycle ergometer, treadmill, walker, etc.), before or after group exercise  Load build-up individually, monitoring via heart rate, blood pressure and Borg scale | 30 |
| **5. Session** | **Stick and ring**  *Location: Rehabilitation center, gymnastics room, size of the playing field approximately 4 x 4 m* |  |
|  | Exploration of the experiences | 10 |
|  | Exercises and games with sticks and rings  *Equipment: stick and rings*  - Gymnastics with sticks, group in circle (to improve mobility, reactive capacity, proprioception).  - Stick grabbing as a game  - Partner games with stick and ring  - Game: "Field clean up" in 2 Teams |  |
|  | Stretching exercise, instruction for self-exercise | 15 |
|  | Farewell | 5 |
|  | **Endurance training** (bicycle ergometer, treadmill, walker, etc.), before or after group exercise  Load build-up individually, monitoring via heart rate, blood pressure and Borg scale | 30 |
| **Optional**  **session** | **Line Dance (optional session instead of ballgames, if participants wish)**  *Location: Rehabilitation center, gymnastics room*  *Equipment: Playlist Line Dance music, boombox* |  |
|  | Exploration of the experiences | 10 |
|  | Learning basic steps without music  Basic steps with music  Learning a dance with basic steps and specials | 30 |
|  | Stretching exercise, instruction for self-exercise | 15 |
|  | Farewell | 5 |
|  | **Endurance training** (bicycle ergometer, treadmill, walker, etc.), before or after group exercise  Load build-up individually, monitoring via heart rate, blood pressure and Borg scale | 30 |
