# Supplementary material for: Hypnotherapy, Intermittent Fasting, and Exercise Group Programs in Atopic Dermatitis: A Randomized Controlled Explorative Clinical Trial During the COVID-19 Pandemic
Source: J Integr Complement Med. 2023 Feb 8;29(2):99–110. doi: 10.1089/jicm.2022.0699 (PMC9942184; doi:10.1089/jicm.2022.0699)
Supplement: Supplemental data [file Suppl_TableS2.docx]

**Supplement Table 2.** Baseline characteristics of all included participants (n = 23).

|  | | N | **HTP**  **(n = 7) Mean ± SD /**  **n (%)** | **IFDP**  **(n = 6) Mean ± SD /**  **n (%)** | **Control**  **(n = 9) Mean ± SD /**  **n (%)** | **Exercise group**  **(n = 4) Mean ± SD /**  **n (%)** | **Total**  **Mean ± SD /**  **(n = 26)**  **n (%)** |
| --- | --- | --- | --- | --- | --- | --- | --- |
| Age (years) | | 23 | 29.2 ± 4.3 | 32.5 ± 14.2 | 39.6 ± 11.5 | 39.0 ± 24.0 | 35.0 ± 12.1 |
| Sex (female) | | 23 | 6 (100) | 5 (83.3) | 8 (88.9) | 1 (50.0) | 20 (87.0) |
| Sex (male) | | 23 | 0 | 1 (16.7) | 1 (11.1) | 1 (50.0) | 3 (13.0) |
| BMI (kg/m²) | | 23 | 21.4 ± 2.6 | 22.0 ± 1.8 | 23.7 ± 3.0 | 23.2 ± 2.7 | 22.6 ± 2.6 |
| German university entrance qualification | | 23 | 6 (100) | 5 (83.3) | 8 (88.9) | 2 (100) | 21 (91.3) |
| Duration of AD (years) | | 23 | 22.2 ± 6.4 | 28.5 ± 16.3 | 38.1 ± 13.0 | 38.0 ± 22.6 | 31.4 ± 14.2 |
| Three Item Severity Score [0-9]* | | 23 | 4.5 ± 1.0 | 4.5 ± 2.2 | 5.9 ± 0.8 | 4.0 ± 1.4 | 5.0 ± 1.5 |
| Concomitant disease | | 23 | 2 (33.3) | 3 (50.0) | 5 (55.6) | 0 | 10 (43.5) |
| Allergic asthma | | 23 | 2 (33.3) | 3 (50.0) | 2 (22.2) | 0 | 7 (30.4) |
| History of Allergy | | 23 | 6 (100) | 4 (66.7) | 7 (77.8) | 2 (100) | 19 (82.6) |
|  | Food allergy | 23 | 4 (66.7) | 3 (50.0) | 3 (33.3) | 2 (100) | 12 (52.2) |
| Influence by climate | | 23 | 6 (100) | 6 (100) | 7 (77.8) | 2 (100) | 21 (91.3) |
| Previous psychotherapy | | 23 | 3 (50.0) | 4 (66.7) | 8 (88.9) | 0 | 15 (65.2) |
| Patients using AD medication (last 3 months) | | 23 | 5 (83.3) | 6 (100) | 9 (100) | 2 (100) | 22 (95.7) |
| Patients applying TCS* last 4 weeks | |  |  |  | 8 (88.9) |  |  |
| Past treatment with hypnotherapy | | 23 | 0 | 1 (16.7) | 2 (22.2) | 0 | 3 (13.0) |
|  | due to AD | 23 | 0 | 0 | 0 | 0 | 0 |
|  | due to other diagnosis | 23 | 0 | 1 (16.7) | 2 (22.2) | 0 | 3 (13.0) |
| Past fasting | | 23 | 4 (66.7) | 2 (33.3) | 5 (55.6) | 0 | 11 (47.8) |
|  | due to AD | 23 | 1 (16.7) | 0 | 2 (22.2) | 0 | 3 (13.0) |
|  | due to other diagnosis | 23 | 3 (50.0) | 2 (33.3) | 3 (33.3) | 0 | 8 (34.8) |
| VAS itching [0-100 mm]^+^ in questionnaire | | 23 | 63.2 ± 18.0 | 57.8 ± 15.6 | 62.1 ± 17.3 | 48.5 ± 7.8 | 60.1 ± 16.1 |
| VAS itching [0-100 mm]^+^ during interview | | 23 | 64.0 ± 14.1 | 56.3 ± 13.9 | 59.9 ± 16.5 | 43.0 ± 22.6 | 58.6 ± 15.6 |
| SCORAD [0-103]* | | 23 | 43.0 ± 13.6 | 38.4 ± 21.0 | 39.1 ± 15.6 | 41.7 ± 26.2 | 40.2 ± 16.3 |
| EASI [0-72]* | | 23 | 12.4 ± 11.0 | 9.4 ± 7.5 | 10.6 ± 8.0 | 14.5 ± 19.9 | 11.1 ± 9.2 |
| VAS skin total AD symptoms [0-100 mm]^++^ | | 23 | 65.7 ± 18.8 | 61.3 ± 23.8 | 59.6 ± 14.7 | 56.5 ± 23.3 | 61.4 ± 17.9 |
| SF-12 physical component scale** | | 22 | 49.8 ± 3.7 | 51.7 ± 8.0 | 50.6 ± 7.8 | 48.0 ± 14.0 | 50.5 ± 7.0 |
| SF-12 mental component scale** | | 22 | 41.7 ± 4.6 | 39.1 ± 10.7 | 37.9 ± 11.1 | 37.0 ± 3.2 | 39.2 ± 8.8 |
| DLQI [0-30]* | | 23 | 12.8 ± 6.3 | 9.0 ± 10.0 | 11.3 ± 6.8 | 13.0 ± 0 | 11.3 ± 7.1 |
| PANAS, positive affect dimension [1-5]* | | 23 | 2.9 ± 0.2 | 3.2 ± 0.4 | 2.7 ± 0.9 | 3.2 ± 0.1 | 2.9 ± 0.6 |
| PANAS, negative affect dimension [1-5]** | | 23 | 2.3 ± 0.8 | 2.1 ± 0.3 | 2.0 ± 0.5 | 1.7 ± 0.2 | 2.1 ± 0.5 |
| Incapacity to work due to AD (8 weeks) | | 23 | 3 (50.0) | 1 (16.7) | 4 (44.4) | 1 (50.0) | 9 (39.1) |
|  | Incapacity to work due to AD (hours/8 weeks) | 9 | 41.7 ± 67.8 | 96 | 55.5 ± 61.5 | 8 | 50.1 ± 55.6 |
| Sleep disturbances due to AD | | 23 | 5 (83.3) | 4 (66.7) | 6 (66.7) | 2 (100) | 17 (73.9) |
|  | VAS sleep disturbances [0-100 mm]^+++^ | 17 | 34.8 ± 19.9 | 51.8 ± 20.8 | 46.0 ± 20.9 | 23.5 ± 6.4 | 41.4 ± 20.2 |
| VAS skin condition [0-100 mm]^++++^ | | 23 | 58.7 ± 27.3 | 51.2 ± 25.9 | 55.9 ± 21.4 | 46.0 ± 5.7 | 54.5 ± 22.5 |
| Patient expectations for HTP [0-10]^#^ | | 23 | 7.2 ± 1.2  median 7.0 | 6.5 ± 2.1  median 7.5 | 5.1 ± 2.4  median 5.0 | 5.5 ± 3.5  median 5.5 | 6.0 ± 2.2  median 7.0 |
| Patient expectations for IFDP [0-10]^#^ | | 23 | 5.7 ± 2.3  median 5.5 | 6.3 ± 2.3  median 7.0 | 6.6 ± 3.2  median 7.0 | 6.5 ± 3.5  median 6.5 | 6.3 ± 2.6  median 7.0 |
| Patient expectations for EP [0-10]^#^ | | 23 | 3.0 ± 2.5  median 2.5 | 4.7 ± 2.2  median 5.5 | 5.0 ± 2.5  median 6.0 | 3.0 ± 1.4  median 3.0 | 4.2 ± 2.4  median 4.0 |

Values are absolute numbers (N), column percentages or means ± standard deviations (SD)

*lower values indicate better status, **higher values indicate better status, ^+^0 = no itching, 100 = extreme itching, ^++^0 = no symptoms, 100 = extreme symptoms, ^+++^0 = no sleep disturbance, 100 = extreme sleep disturbance, ^++++^0 = undisturbed skin, 100 = extremely disturbed skin, ^#^0 = no improvement, 10 = complete recovery,

AD = atopic dermatitis, DLQI = Dermatology Life Quality Index, EASI = Eczema Area and Severity Index, HTP = hypnotherapy group program, IFDP = intermittent fasting with diet adjustments group program, EP = exercise program, MCS = mental component scale, PANAS = Positive and Negative Affect Schedule, PCS = physical component scale, SCORAD = SCORing Atopic Dermatitis, SD = standard deviation, SF-12 = 12-item Short Form Health Survey, TCS = topical corticosteroids class I-III, VAS = visual analogue scale
