# Supplementary material for: Hypnotherapy, Intermittent Fasting, and Exercise Group Programs in Atopic Dermatitis: A Randomized Controlled Explorative Clinical Trial During the COVID-19 Pandemic
Source: J Integr Complement Med. 2023 Feb 8;29(2):99–110. doi: 10.1089/jicm.2022.0699 (PMC9942184; doi:10.1089/jicm.2022.0699)
Supplement: Supplemental data [file Suppl_Data.zip › 12RotterCONSERVEChecklistsCAMATOPII.docx]

# CONSERVE Checklists

Use CONSERVE-CONSORT for completed trial reports and CONSERVE-SPIRIT for trial protocols.

| CONSERVE-CONSORT Extension: 15.08.2022 | | | | | |
| --- | --- | --- | --- | --- | --- |
| Item | Item Title | Description | | | Page No. |
| I. | Extenuating Circumstances | Describe the circumstances and how they constitute extenuating circumstances. | | | 4-5 |
| II. | Important Modifications | 1. Describe how the modifications are important modifications. | | | 4-5 |
|  |  | 1. Describe the impacts and mitigating strategies, including their rationale and implications for the trial. | | | (see below) |
|  |  | 1. Provide a modification timeline. | | | Figure 1 |
| III. | Responsible Parties | State who planned, reviewed and approved the modifications. | | | 4-5,  Figure 1 |
| IV. | Interim data | If modifications were informed by trial data, describe how the interim data were used, including whether they were examined by study group, and whether the individuals reviewing the data were blinded to the treatment allocation. | | | N/A |
| CONSORT Number and Item | | For each row, if important modifications occurred check “direct impact” and/or “mitigating strategy” and describe the changes in the trial manuscript or supplement. Check “no change” for items that are unaffected in the extenuating circumstance. | | | Page No. |
|  |  | No Change | Impact* | Mitigating Strategy** |  |
| 1 | Title and abstract |  |  | x | 1 |
| 2 | Introduction | x |  |  |  |
| 3 | Methods: Trial Design |  | x | x | 4-5, Figure 1 |
| 4 | Methods: Participants |  | x | x | 4-5, Figure 1 |
| 5 | Methods: Interventions |  | x | x | 4-6, Figure 1 |
| 6 | Methods: Outcomes | x |  |  |  |
| 7 | Methods: Sample Size | x |  |  |  |
| 8-10 | Methods: Randomisation |  | x | x | 4-5, Figure 1 |
| 11 | Methods: Blinding | x |  |  |  |
| 12 | Methods: Statistical methods |  | x | x | 8 |
| 13 | Results: Participant flow |  | x | x | 9, Figure 2 |
| 14 | Results: Recruitment |  | x | x | 9, Figure 2 |
| 15 | Results: Baseline data |  | x | x | 9, Tables 1,2, Supplement Table 2 |
| 16 | Results: Numbers analysed |  | x | x |  |
| 17 | Results: Outcomes and estimation |  | x | x | 9, Table 2 |
| 18 | Results: Ancillary analyses | x |  |  |  |
| 19 | Results: Harms | x |  |  |  |
| 20 | Discussion: Limitations |  | x | x | 12-13 |
| 21 | Discussion: Generalisability |  | x | x | 12-13 |
| 23 | Other information: Registration | x |  |  |  |
| 24 | Other information: Protocol | x |  |  |  |
| 25 | Other information: Funding | x |  |  |  |
| *Aspects of the trial that are directly affected or changed by the extenuating circumstance and are not under the control of investigators, sponsor or funder.  **Aspects of the trial that are modified by the study investigators, sponsor or funder to respond to the extenuating circumstance or manage the direct impacts on the trial. | | | | | |

| CONSERVE-SPIRIT Extension: [DATE] | | | | | |
| --- | --- | --- | --- | --- | --- |
| Item | Item Title | Description | | | Page No. |
| I. | Extenuating Circumstances | Describe the circumstances and how they constitute extenuating circumstances. | | |  |
| II. | Important Modifications | 1. Describe how the modifications are important modifications. | | |  |
|  |  | 1. Describe the impacts and mitigating strategies, including their rationale and implications for the trial. | | | (see below) |
|  |  | 1. Provide a modification timeline. | | |  |
| III. | Responsible Parties | State who planned, reviewed and approved the modifications. | | |  |
| IV. | Interim data | If modifications were informed by trial data, describe how the interim data were used, including whether they were examined by study group, and whether the individuals reviewing the data were blinded to the treatment allocation. | | |  |
| SPIRIT Item and Number | | For each row, if important modifications occurred, check one or both of “impact” and/or “mitigating strategy” and describe the changes in the protocol. Check “no change” for items that are unaffected in the extenuating circumstance. | | | Page No. |
|  |  | No Change | Impact* | Mitigating Strategy** |  |
| 1 | Title |  |  |  |  |
| 2 | Trial registration |  |  |  |  |
| 3 | Protocol version |  |  |  |  |
| 4 | Funding |  |  |  |  |
| 5 | Roles and responsibilities |  |  |  |  |
| 6 | Background and rationale |  |  |  |  |
| 7 | Objectives |  |  |  |  |
| 8 | Trial design |  |  |  |  |
| 9 | Study setting |  |  |  |  |
| 10 | Eligibility criteria |  |  |  |  |
| 11 | Interventions |  |  |  |  |
| 12 | Outcomes |  |  |  |  |
| 13 | Participant timeline |  |  |  |  |
| 14 | Sample size |  |  |  |  |
| 15 | Recruitment |  |  |  |  |
| 16 | Allocation |  |  |  |  |
| 17 | Blinding (masking) |  |  |  |  |
| 18 | Data collection methods |  |  |  |  |
| 19 | Data management |  |  |  |  |
| 20 | Statistical methods |  |  |  |  |
| 21 | Data monitoring |  |  |  |  |
| 22 | Harms |  |  |  |  |
| 23 | Auditing |  |  |  |  |
| 24 | Research ethics approval |  |  |  |  |
| 25 | Protocol amendments |  |  |  |  |
| 26 | Consent or assent |  |  |  |  |
| 27 | Confidentiality |  |  |  |  |
| 28 | Declaration of interests |  |  |  |  |
| 29 | Access to data |  |  |  |  |
| 30 | Ancillary and post-trial care |  |  |  |  |
| 31 | Dissemination policy |  |  |  |  |
| 32 | Informed consent materials |  |  |  |  |
| 33 | Biological specimens |  |  |  |  |
| *Aspects of the trial that are directly affected or changed by the extenuating circumstance and are not under the control of investigators, sponsor or funder.  **Aspects of the trial that are modified by the study investigators, sponsor or funder to respond to the extenuating circumstance or manage the direct impacts on the trial. | | | | | |
